# A CRISPR-Cas12a—Based platform for ultrasensitive, rapid, and highly specific detection of *Mycoplasma pneumonia* in clinical application

**DOI:** 10.3389/fbioe.2023.1022066

**Published:** 2023-01-17

**Authors:** Nan Jia, Juan Zhou, Fei Xiao, Baoying Zheng, Xiaolan Huang, Chunrong Sun, Jin Fu, Zheng Xu, Min Chen, Yi Wang

**Affiliations:** ^1^ Experimental Research Center, Capital Institute of Pediatrics, Beijing, China; ^2^ Respiratory Medicine, Capital Institute of Pediatrics, Beijing, China

**Keywords:** *Mycoplasma pneumoniae*, multiple cross displacement amplification, CRISPR, Cas12a, community-acquired pneumonia

## Abstract

*Mycoplasma pneumoniae* (MP), which is responsible for a majority of community-acquired pneumonia (CAP) in children, has been largely underestimated. Here, we coupled multiple cross displacement amplification (MCDA) technique with CRISPR-Cas12a-based biosensing system to design a novel detection platform termed MP-MCDA-CRISPR assay for MP infection diagnosis and clinical application. The MP-MCDA-CRISPR assay amplified the CARDS gene of MP by MCDA method, followed by *trans*-cleavage of the reporter molecular upon the formation of CRISPR-Cas12a-gRNA-target DNA complex, which was confirmed by the release of fluorescent signals. A set of standard MCDA primers, an engineered CP1 primer, a quenched fluorescent ssDNA reporter, and a gRNA were designed targeting the CARDS gene of MP. The optimal temperature for MCDA pre-amplification is 64°C, and the time for CRISPR-Cas12a-gRNA biosensing process is 5 min. The limit of detection (LoD) of the MP-MCDA-CRISPR assay is 50 fg per reaction without any cross-reaction with other non-MP pathogens. The MP-MCDA-CRISPR assay accurately identified the 50 real time-PCR positive clinical samples and 78 negative ones. Taken together, the MP-MCDA-CRISPR assay designed here is a promising diagnostic tool for point-of care (POC) testing of MP infection.

## Introduction

Lower respiratory tract infection has been recognized as a major cause of morbidity and mortality in children, and *Mycoplasma pneumoniae* (MP) was the most commonly detected bacterial pathogen among children <18 years old hospitalized with community-acquired pneumonia (CAP) (Jain et al., 2015). Apart from pneumonia, MP was also associated with upper respiratory infections and a wide range of extrapulmonary manifestations, such as mucositis (Vujic et al., 2015), encephalitis (Daxboeck et al., 2004), and Stevens-Johnson syndrome (SJS) (Olson et al., 2015). Although MP infection was usually considered as mild and self-limited (Waites and Talkington, 2004) for which the burden of MP infection was usually underestimated, it was reported that more than 10% children hospitalized for MP infection were admitted to the ICU (Kutty et al., 2019). Along with the emergence and wide spread of macrolide-resistant MP isolates (Li et al., 2009; Yamada et al., 2012), identification and surveillance of MP infection needs to further strengthen.

Due to the clinical characteristics of MP infection are non-specific (Kutty et al., 2019), identification and confirmation of MP infection is mainly based on laboratory diagnosis. At present, there are three methods commonly used for detection of MP infection, i.e., culture, serology and molecular-based methods. Although culture is the traditional “gold standard” for identification of MP infection (specificity is 100%), long time and fastidious cultivation requirements makes it impractical (Waites et al., 2017). Optimum serological testing of MP infection usually needs two sera specimens collected at least 2 weeks apart (Bajantri et al., 2018), which is time-consuming; besides, insufficient immunological responses of the infant and elderly (Bajantri et al., 2018) and cross-activity with cytomegalovirus or Epstein-Barr virus infections (Medjo et al., 2014) also limit the sensitivity and specificity of MP testing, making this method not readily available. Molecular-based methods have been considered as “new gold standard” credited with their superior sensitivity and time-effectiveness (Waites et al., 2012; Loens and Ieven, 2016; Waites et al., 2017), and a series of commercially diagnostic methods are available for MP infection detection, such as the real-time PCR-based method RespiFinder SMART 22 (Patho Finder, Maastrict, the Netherlands) (Pillet et al., 2013), loop-mediated isothermal amplification (LAMP)-based method *illumigene Mycoplasma* (Meridian BioScience, Inc., Cincinnati, OH, United States) (Ratliff et al., 2014) and strand displacement assays (SDA)-based method BD Probe Tec ET (BD Diagnostics, Sparks, MD, United States) (Loens et al., 2010). Real-time PCRs had lower likelihood of contamination, could provide quantitative data and detect antimicrobial resistance genes (Waites et al., 2017); however, their highly dependence of high-precision apparatus and skilled technicians hampered their widely utilization. LAMPs and SDAs are less expensive and equipment requirement, more easy-to-perform, specific and sensitive than real-time PCR-based methods (Kakuya et al., 2014; Waites et al., 2017), however, the product analysis methods are somewhat labor-consuming or subjective (Li et al., 2020). Therefore, an accurate, rapid, ultrasensitive and highly specific assay for MP detection is sorely needed for case and outbreak identification and treatment and infection prevention guidance.

In recent years, a variety of CRISPR/Cas (clustered regularly interspaced short palindromic repeats and CRISPR associated protein)-related technologies have been harnessed for use in fields of research (Liu et al., 2022), including the nucleic acid detection field (Gootenberg et al., 2017), owing to their merit in terms of efficiency, specificity, and precision for biosensing (Liu et al., 2022). CRISPR/Cas systems are widely distributed in prokaryotic genomes and confer prokaryote acquired immunity against foreign nuclei acids (Barrangou et al., 2007; Brouns et al., 2008; Marraffini and Sontheimer, 2008). The principle of nuclei acid detection with CRISPR/Cas system is based on the collateral cleavage activities of Cas effectors (such as Cas12a, Cas12b, Cas13a, and Cas14), which enable non-specific and indiscriminate cleavage of surrounding non-target single strand RNA and DNA following the guidance of gRNA (guide RNA) (Li et al., 2019a; Li et al., 2019b; Shihong Gao et al., 2021). Up to now, there have been several CRISPR/Cas biosensing systems applied in nuclei acid analysis, such as DETECTR (DNA Endonuclease-Targeted CRISPR Trans Reporter) (Chen et al., 2018), SHERLOCK (Specific High-sensitivity Enzymatic Reporter UnLOCKing) (Gootenberg et al., 2017), HOLMES (One-Hour-Low cost Multipurpose highly Efficient System) (Li et al., 2018b), CRISPR-top (Li et al., 2021) and CRISPR-MCCD, which are proven cost-effective, easily accessible, and widely applicable.

**FIGURE 1:**
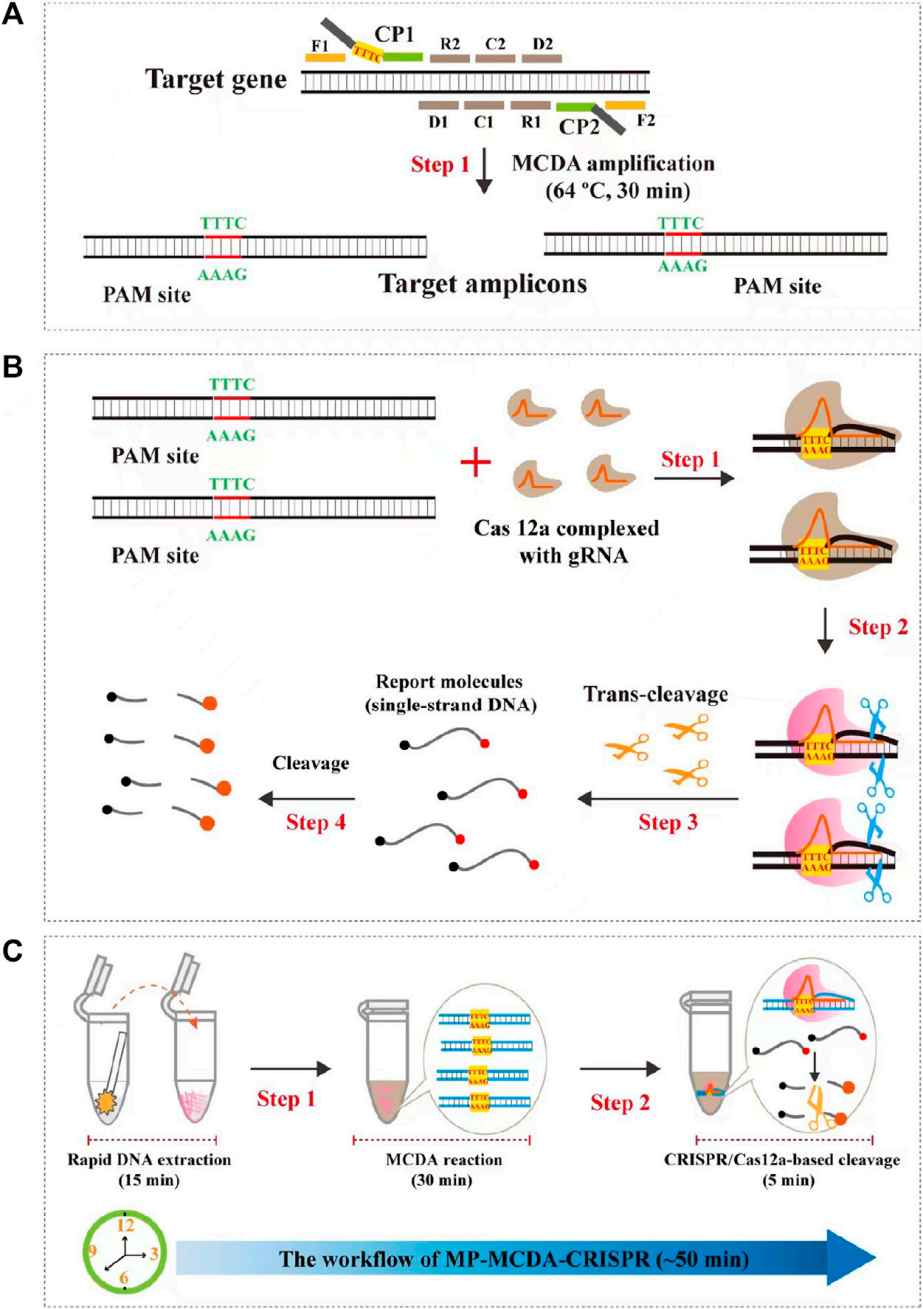
Schematic illustration of the principle of the MP-MCDA-CRISPR assay. **(A)** Schematic illustration of the principle of MCDA with the modified primer. The primer CP1 was modified with a PAM site (TTTC) at the linker region. After amplification, a CRISPR-Cas12a recognition site was constructed in the target amplicons. **(B)** Schematic illustration of the CRISPR-Cas12a detection system. Upon recognition of the target DNA, the CRISPR-Cas12a-gRNA complex cleaves the single stranded DNA reporter molecule and responds with release of fluorescence signal. **(C)** Overview of the MP-MCDA-CRISPR workflow. MP-MCDA-CRISPR assay employs three closely linked steps: DNA extraction (step 1), MCDA pre-amplification (step 2), CRISPR-Cas12a cleavage and data report (step 3). The whole process could be completed in 50 min.

In the current study, we incorporated the CRISPR-Cas12a biosensing system with a relatively new and promising nucleic acid isothermal amplification technology termed multiple cross displacement amplification (MCDA) for timely, accurate, robust, ultrasensitive and highly specific diagnosis of MP infection, and named as MP-MCDA-CRISPR. Furthermore, a protospacer adjacent motif (PAM) site (TTTC) for the CRISPR-Cas12a–related assay was engineered onto the MCDA primers for detecting sequences that lack of PAM sites. Thereafter, we illustrated the principle of the MP-MCDA-CRISPR assay (Figure 1) and validated its feasibility in clinical specimens.

## Materials and methods

### Reagents and instruments

The primers used in this study were synthesized by AOKE Biotech Co., Ltd. (Beijing, China), and the probe and gRNA were by Tianyi-Huiyuan Bioscience & Technology Co., Ltd. (Beijing, China). Genomic DNA kit for nucleic acid extraction and purification was purchased from Beijing TransGen Biotech Co., Ltd. (Beijing, China). The universal isothermal amplification kits, visual detection reagent (VDR), and CRISPR-Cas12a protein (Cpf1) were obtained from HuiDeXin Biotechnology (Tianjin, China). A real-time turbidimeter (LA-320C) was purchased from Eiken Chemical Co., Ltd. (Japan) and the Applied Biosystems^®^ 7500 Real Time PCR System was from ThermoFisher Scientific (United States).

### Bacterial strain and clinical samples

Bacterial strains used in this study were shown in Table 1, including pure cultures of 10 MP strains and 40 other pathogens. Additionally, 128 nasopharyngeal swabs collected from 128 patients suspected of MP infection in the capital institute of pediatrics (CIP) were employed in this study as well for clinical feasibility assessment. Nucleic acid of all the pure cultures and clinical samples were extract and purified by using the genomic DNA kit.

**TABLE 1:** The strains used in this study.

Strains	Source of strains^a^	No. of strains	MP-MCDA-CRISPR^b^
*M. pneumoniae*	Isolated strains (CIP)	10	P
*Bacillus cereus*	Isolated strains (CDC)	1	N
*Citrobacter*	Isolated strains (CDC)	2	N
*Corynebacterium striatum*	Isolated strains (CDC)	1	N
*Enteroadherent Escherichia coli*	Isolated strains (CDC)	1	N
*Enterococcus faecalis*	Isolated strains (CDC)	2	N
*Enteroinvasive E. coli*	Isolated strains (CDC)	1	N
*Enteropathogenic E. coli*	Isolated strains (CDC)	1	N
*Enterotoxic E. coli*	Isolated strains (CDC)	1	N
*Klebsiella pneumoniae*	Isolated strains (CDC)	2	N
*Listeria innocua*	Isolated strains (CDC)	1	N
*Listeria ivanovii*	Isolated strains (CDC)	1	N
*Listeria monocytogenes*	Isolated strains (CDC)	1	N
*Monilia albican*	Isolated strains (CDC)	2	N
*Moraxella catarrhalis*	Isolated strains (CDC)	1	N
*Mycobacterium tuberculosis*	Isolated strains (CDC)	2	N
*Mycoplasma hominis*	Isolated strains (CDC)	2	N
*Mycoplasma penetrans*	Isolated strains (CDC)	2	N
*Mycoplasma primatus*	Isolated strains (CDC)	2	N
*Mycoplasma genitalium*	Isolated strains (CDC)	2	N
*Mycoplasma urealytium*	Isolated strains (CDC)	3	N
*Neisseria meningitidis*	Isolated strains (CDC)	1	N
*Nocardia asteroides*	Isolated strains (CDC)	1	N
*Pseudomonas aeruginosa*	Isolated strains (CDC)	1	N
*Salmonella* sp.	Isolated strains (CDC)	2	N
*Shiga toxin-producing E. coli*	Isolated strains (CDC)	1	N
*Shigella baumannii*	Isolated strains (CDC)	1	N
*Shigella sonnei*	Isolated strains (CDC)	1	N
*Staphylococcus amber*	Isolated strains (CDC)	1	N
*Staphylococcus epidermidis*	Isolated strains (CDC)	1	N
*Staphylococcus haemolyticus*	Isolated strains (CDC)	2	N
*Stenotrophomonas maltophilia*	Isolated strains (CDC)	1	N
*Steptococcus salivarius*	Isolated strains (CDC)	1	N
*Streptococcus aureus*	Isolated strains (CDC)	1	N
*Streptococcus pneumoniae*	Isolated strains (CDC)	1	N
*Streptococcus pyogenes*	Isolated strains (CDC)	1	N
*Streptococcus suis*	Isolated strains (CDC)	2	N

^a^ CIP, Capital Institute of Pediatrics; CDC, Chinese center for disease control and prevention.

^b^ P, positive; N, negative.

### Design of multiple cross displacement amplification primers and gRNA

**FIGURE 2:**
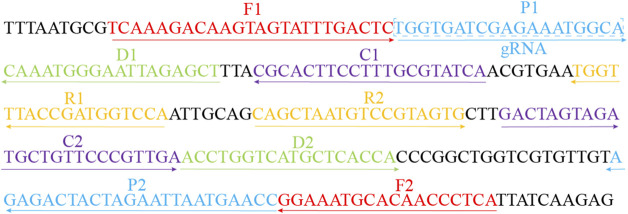
Sequences and locations of the CARDS gene of MP used to design the MCDA primers and gRNA. Locations of MCDA primers are underlined and gRNA is in the box. The right arrow and left arrow represent the sense and complementary sequence used in this study, respectively.

According to the principle of MCDA assay, the primers of MP were designed based on the CARDS gene (community-acquired respiratory distress syndrome toxin gene) by using the PRIMER PREMIER 5.0 software. BLAST analysis was used to verify the specificity of MP-MCDA primers. In addition, the gRNA of MP CARDS gene was designed according to the CRISPR-Cas12a detection mechanism. The positions of MCDA primers and gRNA are shown in Table 2 and Figure 2. PAM sites (TTTC) were added into the linker region of the regular CP1 primer of MCDA for CRISPR-Cas12a biosensing. The principle of analysis based on MCDA and CRISPR-Cas12a is shown in Figure 1. The MCDA primers, gRNA and probe sequences are shown in Table 2.

**TABLE 2:** The primers, probe, and gRNAs used in the current study.

Primers/Probes/gRNA	Sequence and modification (5′-3′)^a^	Length^b^
MCDA primers		
F1	TCA​AAG​ACA​AGT​AGT​ATT​TGA​CTC	24 nt
F2	TGAGGGTTGTGCATTTCC	18 nt
CP1	TGA​TAC​GCA​AAG​GAA​GTG​CG**TTT​C**TGG​TGA​TCG​AGA​AAT​GGC​A	43 nt
CP2	GAC​TAG​TAG​ATG​CTG​TTC​CCG​TTG​AGG​TTC​ATT​AAT​TCT​AGT​AGT​CTC​T	49 nt
C1	TGA​TAC​GCA​AAG​GAA​GTG​CG	20 nt
C2	GAC​TAG​TAG​ATG​CTG​TTC​CCG​TTG​A	25 nt
D1	AAG​CTC​TAA​TTC​CCA​TTT​G	19 nt
D2	ACCTGGTCATGCTCACCA	18 nt
R1	TGGACCATCGGTAAACCA	18 nt
R2	CAGCTAATGTCCGTAGTG	18 nt
gRNA	UAA​UUU​CUA​CUA​AGU​GUA​GAU​UGG​UGA​UCG​AGA​AAU​GGC​A	40 mer
Probe^c^	FAM-TATTATTATTATTATTT-BHQ1	17 mer

^a^ CP1 primer was modified in linker region with a PAM site (TTTC).

^b^ nt, nucleitide; mer, monomeric unit.

^c^ 5′end of probe was labeled with FAM, and 3′end was labeled with BHQ1.

### Multiple cross displacement amplification

The pre-amplification step of MCDA was performed with an isothermal amplification kit as per the manufacturer’s instructions (HuiDeXing Biotech. Co., Ltd., Tianjing, China). The reaction system of MP-MCDA was 25 μL, including 12.5 μL of 2× reaction buffer, 0.4 μM each of F1 and F2, 1.6 μM each of CP1 and CP2, 0.8 μM each of C1, C2, D1, D2, R1, and R2, 1.0 μL of Bst 2.0 DNA polymerase (8 U), 1 μL (pure bacteria) or 5 μL (clinical sample) DNA template, and appropriate volume of distilled water (DW) to 25 μL. The reaction process was conducted with the real-time turbidity (LA-320C) to monitor the amplification results and optimize the amplification temperature.

### CRISPR-Cas12a based assay

In this study, Cas12a (Cpf1) was used for CRISPR-Cas-based *trans*-cleavage detection. The CRISPR-Cas12a-gRNA complex was formed by incubating 100 nM CRISPR-Cas12a (Cpf1) (Cat No: 10148305) with 100 nM gRNA in 2× HDX buffer at 37°C for 10 min. Then, the complex should be used immediately or stored at low temperature (0°C–4°C) for no more than 12 h.

The CRISPR-Cas12a-based *trans*-cleavage assay was conducted in a 100 μL mixture, including 2 μL of MCDA products, 2.5 μL of probe, 18 μL of CRISPR-Cas12a-gRNA complex, 50 μL of 2× HDX buffer, and 27.5 μL of DW, following a process of incubation at 37°C for 10 min. The results were monitored by real-time fluorescence (RTF) method, with the probe (5′-Fam-TATTATTATTATTATTT-BHQ1-3′, 10 μM) utilized for CRISPR-Cas12a *trans*-cleavage detection (Zhu et al., 2021).

### Sensitivity and specificity of the MP-MCDA-CRISPR assay

To verify the specificity of MP-MCDA-CRISPR assay, a total of 10 MP strains and 40 non-MP strains were used (Table 1), with distilled water (DW) applied as the blank control. Nucleic acid of all the strains were extracted and purified by using the Beijing TransGen Genomic DNA kit. In addition, nucleic acids of MP strains were also serially diluted from 5 ng to 5 pg per reaction with 10-fold attenuation for sensitivity analysis. In both kind of analysis, the MP-MCDA-CRISPR assay was performed as described above, and the results were reported by both RTF and VDR methods. Each test was repeated triple.

### Validation of the feasibility of MP-MCDA-CRISPR assay in clinical samples

To further verify the feasibility of MP-MCDA-CRISPR assay in clinical settings, we collected 128 nasopharyngeal swabs suspected of MP infection based on clinical symptoms from CIP. The MP-MCDA-CRISPR assay was conducted as described above. All the clinical specimens were tested by real-time PCR method as well. Results of both methods were compared. Particularly, all the procedures were approved by the ethics committee of CIP, and all the samples obtained the informed consent signed by the participant’s guardian.

## Results

### Overview of MP-MCDA-CRISPR detection system

The principle of MP-MCDA-CRISPR assay is shown in Figure 1. Firstly, the extracted MP DNA templates were exponentially pre-amplificated by the MCDA method within 30 min, of which the core primer CP1 was engineered with a PAM site (TTTC) for Cas12a effector at its linker region (Figure 1A). Then the gRNA, which formed a complex with CRISPR-Cas12a effector (Figure 1B, step 1), guided Cas12a molecules to recognize the target amplicons by the PAM site (Figure 1B, step 2). Finally, the trans-cleavage activity of the CRISPR-Cas12a effector was activated and the single-strand DNA (ssDNA) reporter molecules (5′-FAM-TATTATTATTATTATTT-BHQ1-3′) were ultrafast digested (Figure 1B, step 3, step 4), which was detected by the Applied Biosystems^®^ 7500 Real Time PCR System. For the negative results, the ssDNA reporting molecules were not digested and the fluorescence signals could not be detected.

### Optimal reaction conditions for MP-MCDA-CRISPR assay

**FIGURE 3:**
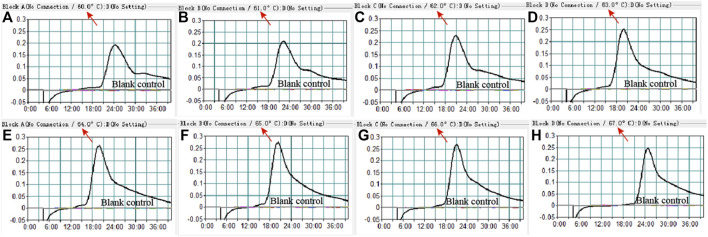
Temperature optimization for the MCDA assay. The MCDA method was used to detect pre-amplification of the CARDS gene of MP. The dynamic curves **(A–H)** were obtained at different temperatures ranging from 60°C to 67°C, with 64°C as the optimal MCDA reaction temperature for MP testing.

**FIGURE 4:**
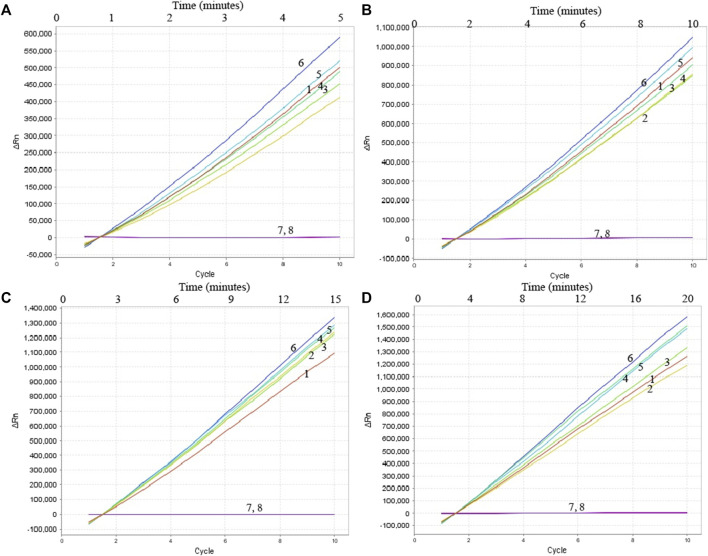
Optimization of reaction time of CRISPR-Cas12a-based biosensing system. Real-time fluorescence detection was used for reporting the CRISPR-Cas12a-gRNA cleavage results at 5 min **(A)**, 10 min **(B)**, 15 min **(C)**, and 20 min **(D)**. Stable fluorescence signal can be detected within 5 min. Curves 1–7, the fluorescence signal of the MP-MCDA reaction products yield from 5 ng, 500 pg, 50 pg, 5 pg, 500 fg, 50 fg, and 5 fg of MP genomic DNA; Curve 8, fluorescence signal of the blank control (DW).

To confirm the optimal temperature of MP-MCDA, temperatures ranging from 60°C to 67°C with an interval of 1°C were tested. As shown in Figure 3, 64°C was detected to be the optimal reaction temperature for the MP-MCDA reaction. Afterwards, the reaction time for CRISPR-Cas12a detection step was also optimized by comparing the results at 5, 10, 15, 20 min, respectively. As shown in Figure 4, a reaction time of 5 min was recommended for the CRISPR-Cas12a cleavage step of MP-MCDA-CRISPR assay.

### Specificity of MP-MCDA-CRISPR assay

**FIGURE 5:**
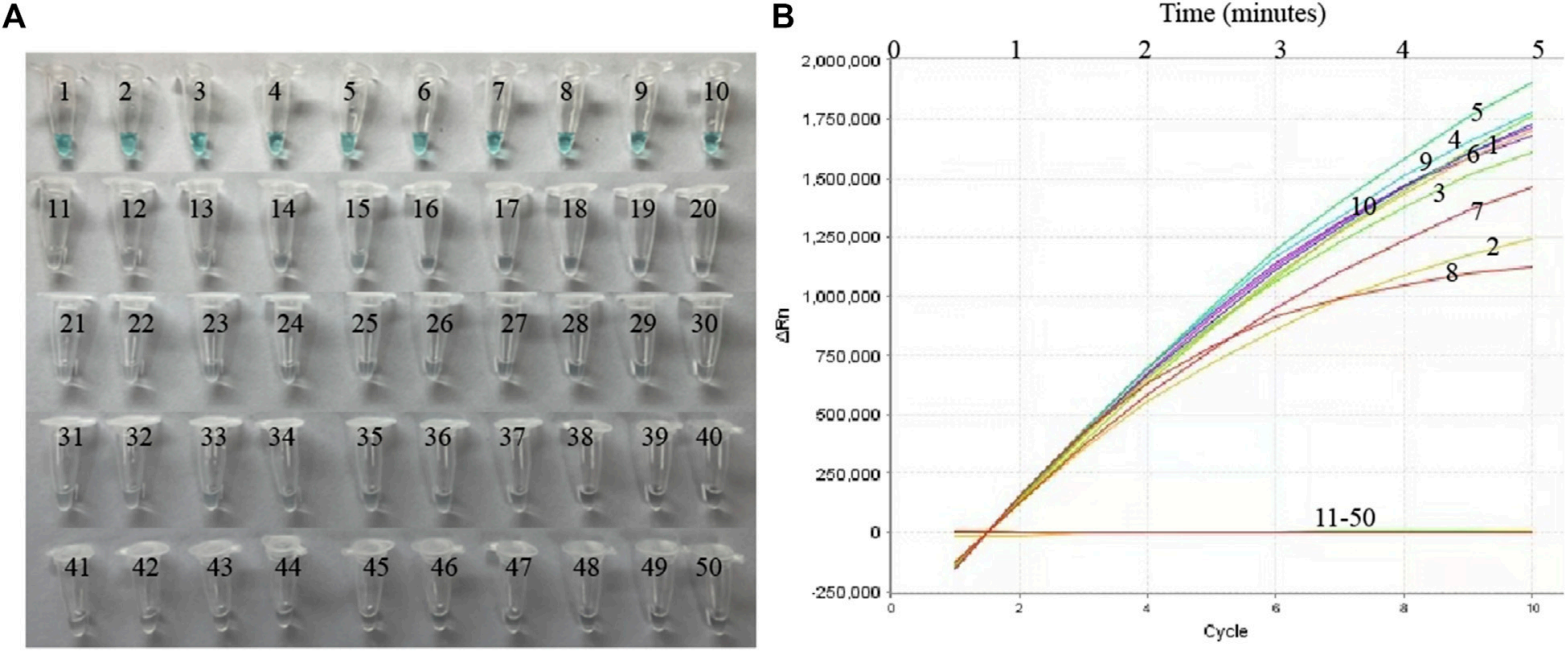
The specificity of the MP-MCDA-CRISPR assay. The specificity of the MP-MCDA-CRISPR assay reported by VDR method **(A)** and RTF method **(B)**, respectively. Tubes/Curves 1–10 represent the MP agents, and Tubes/Curves 11–50 represent non-MP pathogens.

Nucleic acid templates extracted from 10 MP and 40 non-MP strains were used to evaluate the specificity of the MP-MCDA-CRISPR assay. The MP-MCDA-CRISPR was conducted under optimal reaction conditions, and the results were further confirmed using visual MCDA technique (VDR-based method). Readouts of both methods demonstrated that the 10 MP strains were positive, while all the non-MP strains were negative (Figure 5), implying that the specificity of MP-MCDA-CRISPR assay was 100%. Therefore, the MP-MCDA-CRISPR assay designed in this study has a high selectivity to the target nucleic acid.

### Sensitivity of MP-MCDA-CRISPR assay

**FIGURE 6:**
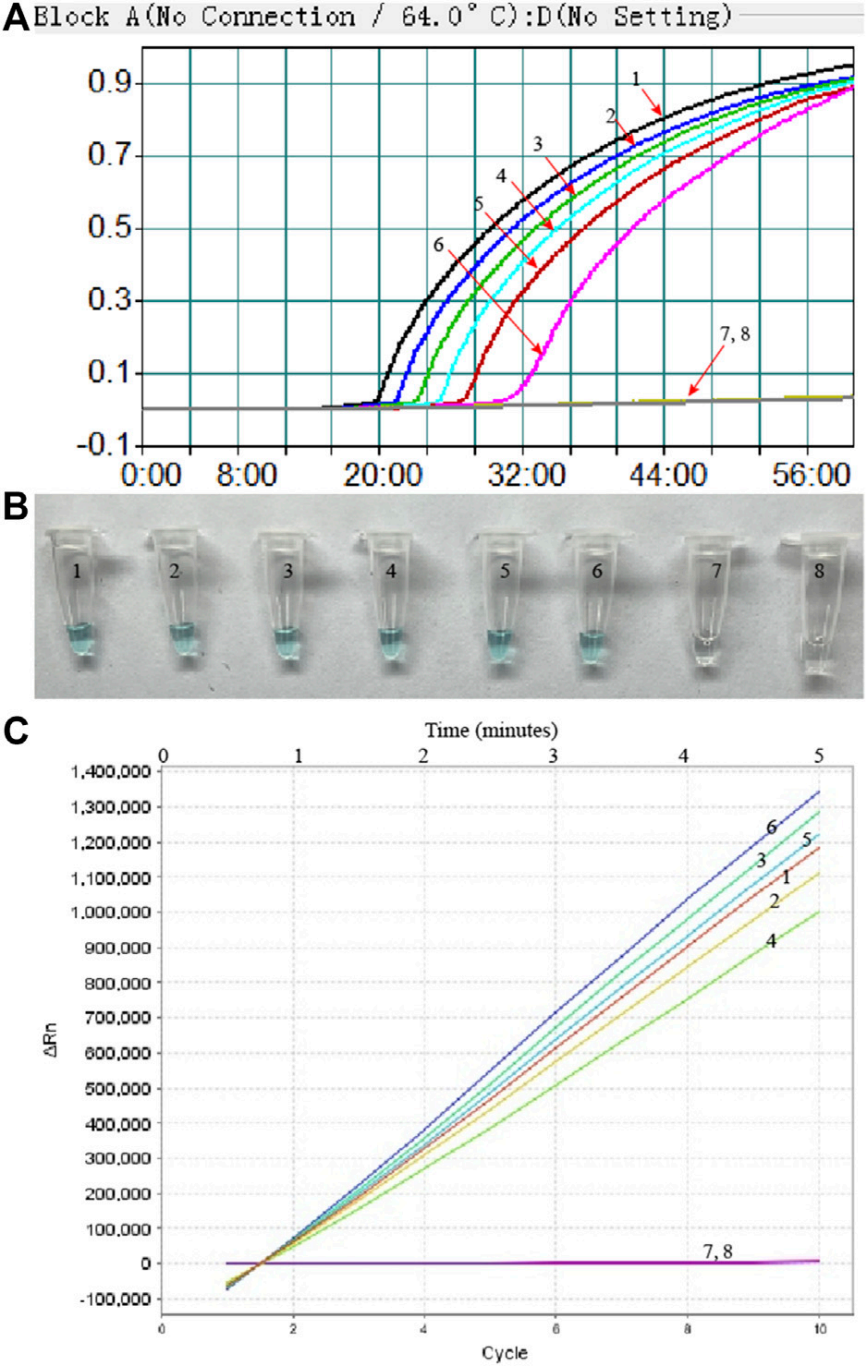
The sensitivity of the MP-MCDA-CRISPR assay. Real-time turbidity **(A)**, VDR **(B)** and RTF **(C)** methods were used to report the MP-MCDA-CRISPR assay results, respectively. Curves/Tubes 1–8 represent the MP genomic DNA concentrations of 5 ng, 500 pg, 50 pg, 5 pg, 500 fg, 50 fg, and 5 fg per reaction, and the blank control (DW), respectively. The LoD of the MP-MCDA-CRISPR assay was 50 fg per reaction.

To evaluate the sensitivity of MP-MCDA-CRISPR assay, the serially diluted MP DNA (ranging from 5 ng to 5 fg per microliter) were used. As shown in Figure 6, readouts of the real-time turbidity, CRISPR and VDR methods indicated that the limit of detection (LoD) of MP-MCDA-CRISPR assay was 50 fg per reaction.

### Feasibility of MP-MCDA-CRISPR assay in clinical samples

**FIGURE 7:**
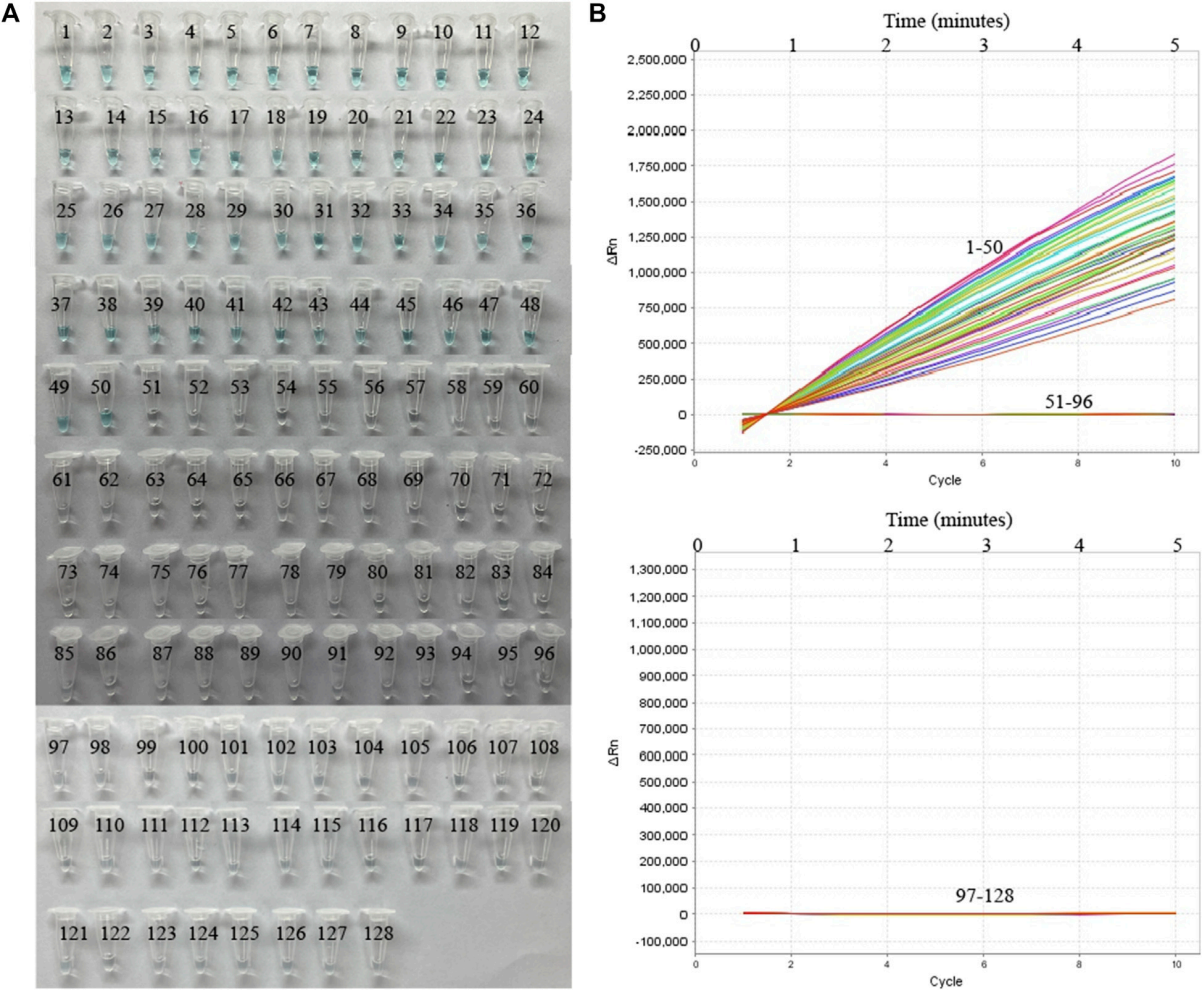
MP-MCDA-CRISPR results of 128 clinical samples suspected of MP infection. VDR **(A)** and RTF **(B)** methods were used to report the MP-MCDA-CRISPR assay results, respectively. Tubes/Curves 1–50 represent the 50 real-time PCR positive samples; Tubes/Curves 51–128 represent the 78 real-time PCR negative samples.

To further verify whether MP-MCDA-CRISPR assay could be used for MP detection in clinical samples, 128 nasopharyngeal swab samples suspected of MP infection were simultaneously detected by MP-MCDA-CRISPR and RT-PCR methods. Identical to the RT-PCR results, the 50 positive samples were confirmed as MP infection by MP-MCDA-CRISPR assay, while the 78 negative ones were considered as non-MP infection likewise (Figure 7), suggesting a consistency of 100% between the two methods (Table 3). These results suggested that the MP-MCDA-CRISPR assay established in this study could be used as a valuable technique for detecting MP infection in clinical settings.

**TABLE 3:** Results comparison of MP-MCDA-CRISPR assay and real-time PCR for MP detection in clinical specimens.

Detection assay	Nasopharyngeal swab samples	
	Positive	Negative
MP-MCDA-CRISPR	50 (39%)	78 (61%)
Real-time PCR	50 (39%)	78 (61%)

## Discussion

Here, a novel MP-MCDA-CRISPR assay, which combined the CRISPR-Cas12a-based biosensing system with MCDA, was established and evaluated for rapid detection of MP infection in clinical settings. Hitherto, various isothermal amplification techniques coupled with different biosensing systems have been developed for MP infection diagnosis, such as MCDA combined with nanoparticle-based lateral flow biosensor (LFB) (Wang et al., 2019), LAMP combined with LFB (Xiao et al., 2022), and recombinase polymerase amplification (RPA) combined with real-time fluorescent probe (Jiang et al., 2022), incarnating the advantages of sensitivity, specificity, simplicity and rapidity. Discovery of the CRISPR-Cas system provided a superior alternative for nucleic acid detecting platform for the high specificity (single base pair specificity) and sensitivity (only several copies) (Zhu et al., 2021), and the CRISPR-Cas9, CRISPR-Cas12, and CRISPR-Cas13 systems had been applied for detection of a variety of pathogens, such as hepatitis B virus (Chen et al., 2021) and SARS-CoV-2 (Zhu et al., 2021). In this report, the CRISPR-Cas12a system was first applied as the biosensing platform for MP detection for its highly sensitive, specific, and accurate. Particularly, the CP1 primer of standard MCDA method was modified by adding an engineered PAM site (TTTC) at the linker region (Figure 1) to circumvent the limitation of lack of PAM site which is essential for CRISPR-Cas12a-based biosensing platforms. Thus, the newly developed MCDA-CRISPR assay could detected any target sequences which were dearth of PAM sites.

An ideal laboratory diagnostic technique should be rapid, specific, sensitive, and easy to operate. In order to improve the sensitivity of MP-MCDA-CRISPR detection, CARDS gene of MP was pre-amplified by MCDA method. MCDA is a relatively new isothermal amplification technique which is independence of complex and expensive thermal cycling instruments and skilled personnel (Wang et al., 2015). The MCDA method contained a set of 10 primers extending 10 different regions of the target gene, i.e., a pair of replacement primers (F1 and F2), a pair of cross primers (CP1 and CP2) and three pairs of amplification primers (C1, C2, D1, D2, R1, and R2) (Figure 1A), endowing this method a high specificity (Wang et al., 2015). Another great virtue of MCDA assay is its high amplification efficiency, for which only within 30 min and minor amounts of templates (as low as 50 fg) the MCDA assay could reach to the cut-off value of detection (Zhang et al., 2020). More importantly, the MCDA-CP1 primer was engineered with a CRSIPR-Cas12a PAM site (TTTC), which played an important role for location of CRISPR-Cas12a-gRNA complex to the specific amplicons (Figure 1). Hence, the target gene located in the genome of MP could be sensitively, timely and accurately detected by the MP-MCDA-CRISPR assay.

In this study, the MCDA products were detected by the CRISPR-Cas12a-based biosensing system. For this biosensing system, the pivotal component is the CRISPR-Cas12a effector, which could perform collateral cleavage on non-targeted ssDNA upon the formation of the CRISPR-Cas12a-gRNA-target DNA ternary complex following the guidance of gRNA to the target DNA (Li et al., 2018a). Besides, a quenched fluorescent ssDNA reporter (FAM-TATTATTATTATTATTT-BHQ1) was applied as the probe, which released fluorescent signals upon *trans*-cleaved by the Cas12a effector (Figure 1B). The RTF method was used in this study for reporting the MP-MCDA-CRISPR outcomes. In positive reactions, the reporter molecule was cleaved and the produced fluorescent signals was captured by a real-time fluorescent detector. While in the negative reactions, none fluorescent signal was detected for the failure of cleavage of reporter molecule owing to the lack of target DNA. Last but not least, the newly devised MP-MCDA-CRISPR assay was specific enough and had no cross-reaction with other non-MP pathogens.

The newly devised MP-MCDA-CRISPR assay was verified for clinical feasibility as well. The 50 real-time quantitative PCR-positive samples were tested positive by the MP-MCDA-CRISPR assay, so did the 78 real-time quantitative PCR-negative samples as negative in MP-MCDA-CRISPR assay. The concordance between the MP-MCDA-CRISPR assay and the real-time quantitative PCR test demonstrated the reliability of MP-MCDA-CRISPR assay in MP detection for clinical application. The whole detection procedure, including a 15 min process for genomic DNA template preparation, a 30 min process for MCDA pre-amplification, and a 5 min process for CRISPR-Cas12a-gRNA detection and results readout, could be completed within 50 min (Figure 1C). To sum up, with the advantages of low degree of instrument dependence, rapid turnaround time, and accurate detection capability, the MP-MCDA-CRISPR assay is expected to become point of care (POC) diagnosis method for MP infection detection, especially in the low-income countries and regions in the world. Further optimization for this newly proposed MP-MCDA-CRISPR assay could be devising a readout system simpler and easier to perform.

Taken together, we combined MCDA method and CRISPR-Cas12a-based biosensing system to develop a novel assay called “MP-MCDA-CRISPR” for the sensitive, specific and rapid detection of MP infections. The MP-MCDA-CRISPR assay could detect as low as 50 fg genomic DNA of MP and had no cross-reaction with other pathogens. The whole test could be completed within 50 min without complicated instruments and experienced technicians. Herein, the newly developed MP-MCDA-CRISPR assay is expected to become an important POC method for MP infection detection in clinical settings, especially in the resource-limited areas.

## Data Availability

The raw data supporting the conclusion of this article will be made available by the authors, without undue reservation.
